# Supplementary material for: Engineering programmable CAR and antigen pairing via drug-gated light activation
Source: Nat Commun. 2026 Mar 19;17:4252. doi: 10.1038/s41467-026-70855-9 (PMC13168450; doi:10.1038/s41467-026-70855-9)
Supplement: Supplementary file 1 — Supplementary Information [file 41467_2026_70855_MOESM1_ESM.pdf]

Supplementary Information

Engineering Programmable CAR and Antigen Pairing via Drug-gated Light Activation

Ziliang Huang†, Praopim Limsakul, Yiqian Wu, Tianze Guo, Yuxuan Wang, Zhuohang Wu, Linshan Zhu, Molly E. Allen, Longwei Liu, Yingxiao Wang*

† First author. * Corresponding author: Yingxiao Wang, ywang283@usc.edu.

Supplementary Fig. 1

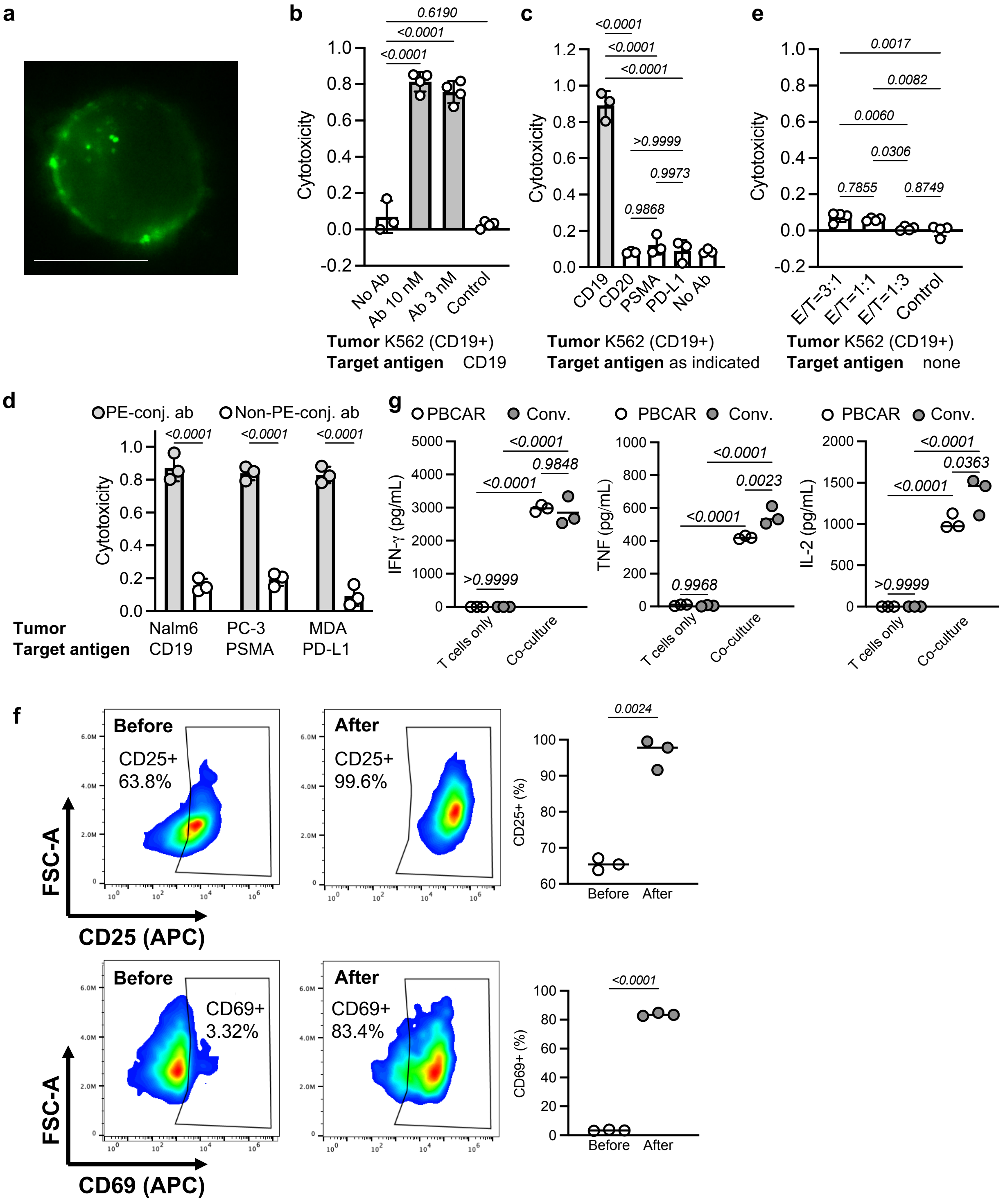

Supplementary Figure 1. PEbody PAT CAR can be programmed to target different antigens and cell types. (a) Surface expression of PEbody CAR (PEbody CAR-GGSGGT-eGFP) on primary T cells. Scale bar, 10 μm. **(b)** Cytotoxicity of PEbody PAT CAR T cells against K562 (CD19+) tumor cells with PE-conjugated CD19 antibody. Control, co-culture without T cells. E/T = 3:1, *n* = 3 biologically independent samples. Data are presented as mean values +/- SD; one-way ANOVA with Sidak's multiple comparisons test. **(c)** Comparison of cytotoxicity with different PE-conjugated antibodies. K562 (CD19+) cells were co-cultured with PEbody PAT CAR T cells with different PE-conjugated antibodies as indicated. E/T = 3:1, *n* = 3 biologically independent samples. Data are presented as mean values +/- SD; one-way ANOVA with Sidak's multiple comparisons test. **(d)** Comparison of cytotoxicity between antibodies with or without PE-conjugation. E/T = 1:5 (Nalm6), 1:1 (PC-3, MDA), *n* = 3 biologically independent samples. Data are presented as mean values +/- SD; one-way ANOVA with Sidak's multiple comparisons test. **(e)** Basal levels of cytotoxicity (without antibody) of PEbody PAT CAR T cells against K562 (CD19+) cells. Control, co-culture without T cells, *n* = 4 biologically independent samples. Data are presented as mean values +/- SD; one-way ANOVA with Sidak's multiple comparisons test. **(f)** Activation marker characterization. Representative flow plots (left/middle) and quantification (right) of CD25 (top) and CD69 (bottom) of PEbody CAR T cells before and after co-culture with Nalm-6 plus anti-CD19 (PE). Data are presented as mean values ± SD; *n* = 3 biologically independent samples; two-tailed Student's t-test. **(g)** Quantification of secreted cytokines before and after co-culture. Data are presented as mean values ± SD; *n* = 3 biologically independent samples; two-tailed Student's t-test. White, PEbody CAR; grey, conventional CAR. Source data are provided as a Source Data file.

Supplementary Fig. 2

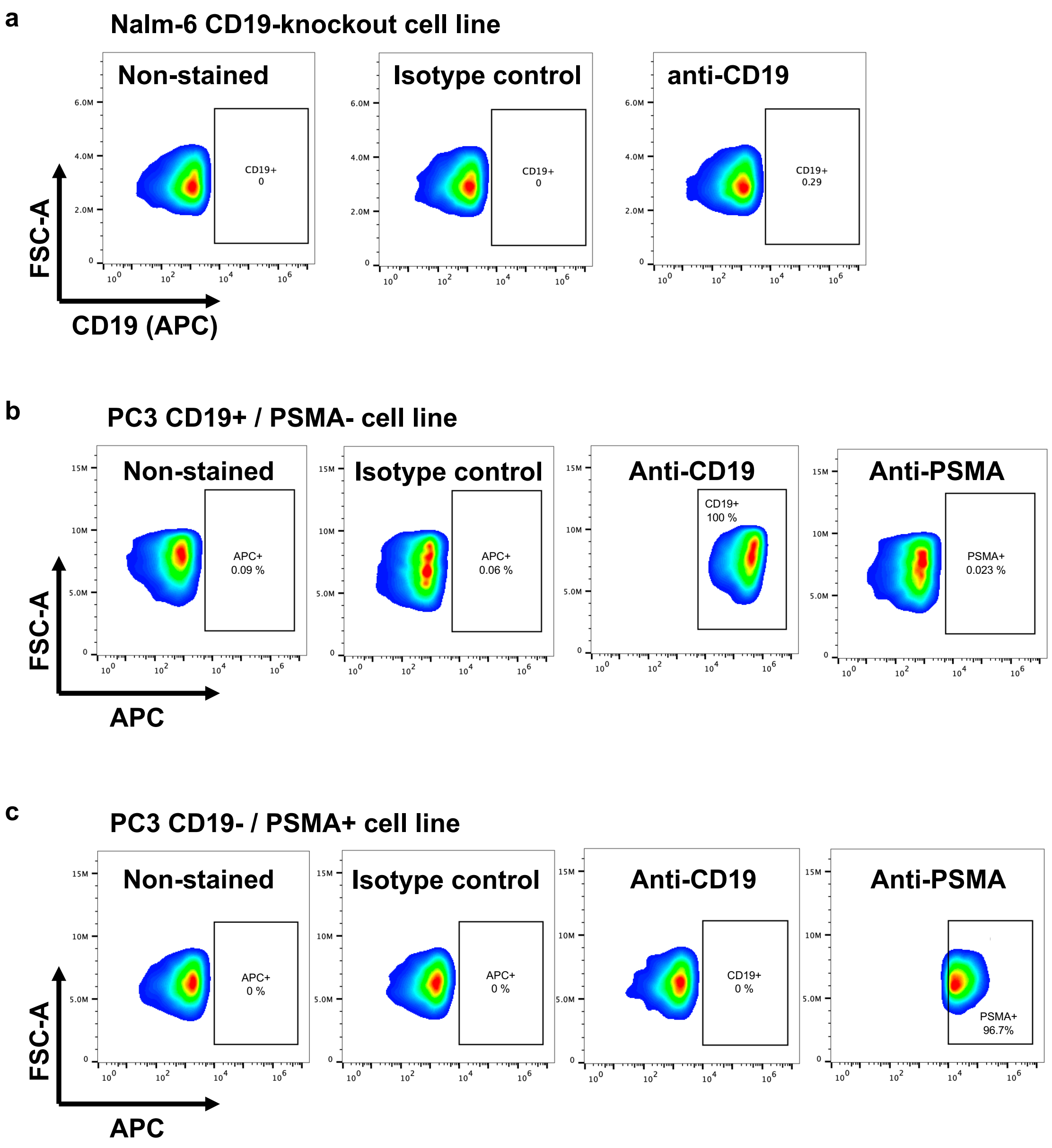

Supplementary Figure 2. Flow cytometry validation of CD19 and PSMA expression in control and engineered cell lines. (a) Nalm-6 CD19-knockout cells show no detectable CD19 staining (non-stained and isotype controls shown). **(b)** PC-3 CD19+/PSMA- line: robust CD19 staining (~100% APC+) and negligible PSMA signal (~0.02% APC+). **(c)** PC-3 CD19-/PSMA+ line: no CD19 staining (0% APC+) and strong PSMA expression (~96.7% APC+).

Supplementary Fig. 3

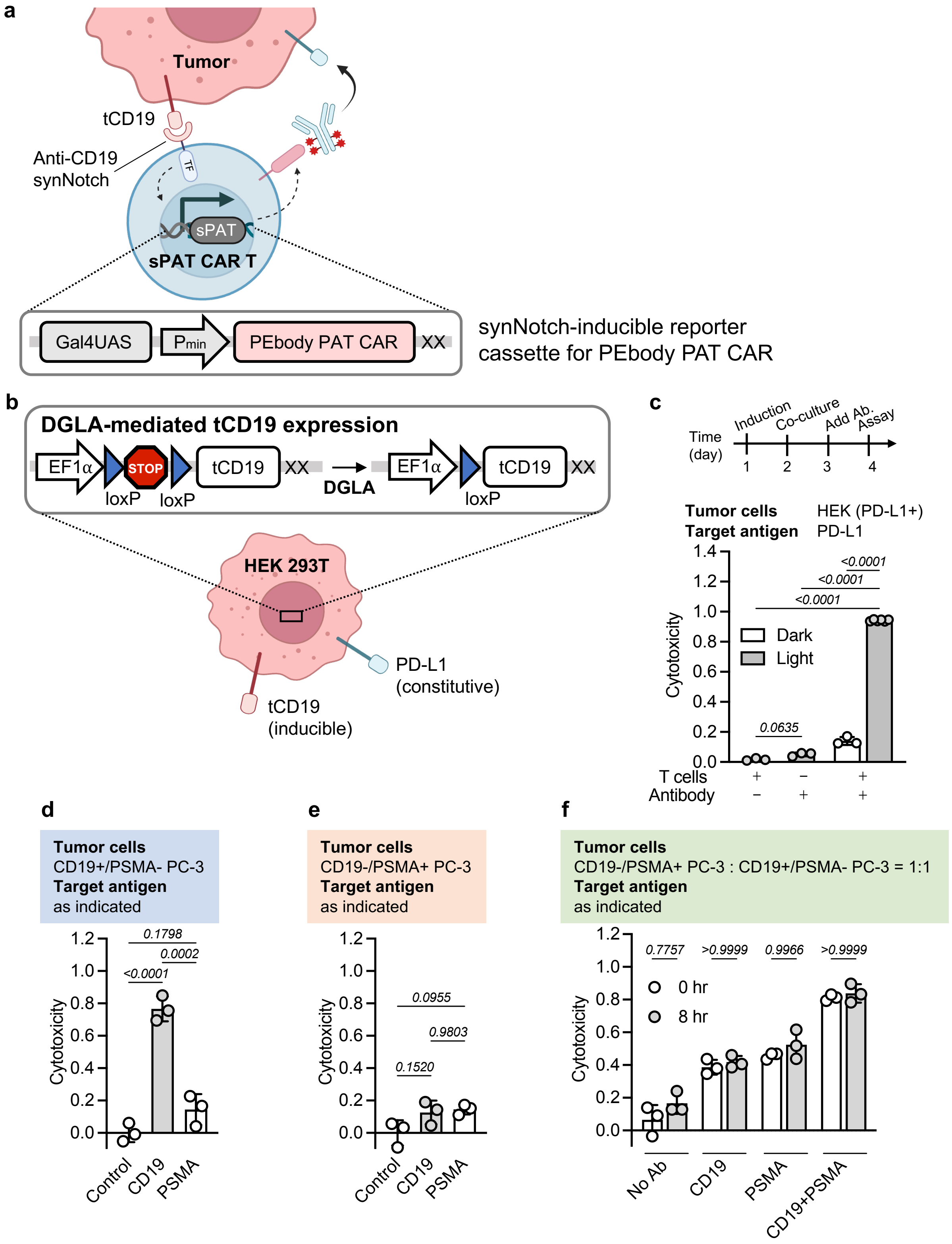

Supplementary Figure 3. Tumor killing via DGLA-sPAT CAR T cells. (a) Schematics showing the tumor killing mechanism of DGLA-inducible antigen and synNotch-mediated PAT CAR approach (DGLA-sPAT). Once the synNotch is activated by DGLA-inducible tCD19, the cleaved Gal4-VP64 (TF) can translocate into nucleus, bind to Gal4UAS, and trigger the PEbody PAT CAR expression on the T cell surface (sPAT CAR), which can be programmed to target the desired antigen(s) on the tumor surface. Created in BioRender. Guo, T. (2026) https://BioRender.com/jt3fjrl. **(b)** The engineered HEK 293T (HEK) cell line for demonstration of DGLA-sPAT CAR-mediated killing. The HEK cells were engineered with the DGLA, inducible tCD19 reporter, and constitutive PD-L1 mimicking the antigen endogenously expressed by the whole tumor population. While the inducible antigen tCD19 can activate sPAT CAR T cells, the constitutive antigen PD-L1 would serve as target for PEbody PAT CAR-mediated cytotoxicity. Created in BioRender. Guo, T. (2026) https://BioRender.com/4hplh4z. **(c)** Tests of DGLA-sPAT CAR approach for tumor killing. Upper panel, experiment timeline. HEK 293T cells (engineered as PD-L1+) engineered with DGLA and inducible tCD19 reporter were seeded on day 0. Cells were then stimulated by blue light to induce tCD19 expression (day 1). The HEK cells after the indicated treatment were co-cultured with sPAT CAR T cells (day 2). PE-conjugated PD-L1 antibody was later added for sPAT CAR-mediated killing. Luciferase assays were performed 24 hr after antibody adding. Lower panel, cytotoxicity of DGLA-sPAT CAR approach under different conditions. T cells (+/−), with or without sPAT CAR T cells; antibody (+/−), with or without antibody addition; light/dark, with or without DGLA induction. E/T = 1:1, n = 3 biologically independent samples. Data are presented as mean values +/- SD; two-way ANOVA with Sidak's multiple-comparisons test. **(d-e)** Cytotoxicity of sPAT CAR T cells against engineered PC-3 cells expressing either CD19 (**d**) or PSMA (**e**). Control, co-culture without T cells; CD19, with PE-conjugated CD19 antibody; PSMA, with PE-conjugated PSMA antibody; E/T = 1:1, n = 3 biologically independent samples. Data are presented as mean values +/- SD; one-way ANOVA with Sidak's multiple-comparisons test. **(f)** Cytotoxicity of sPAT CAR T cells against heterogeneous PC-3 cells expressing either CD19 (CD19+/PSMA-, 50 %) or PSMA (CD19-/PSMA+, 50%). Antibody adding was performed at 0 hr or 8 hr after co-culture (as indicated). CD19, with PE-conjugated CD19

antibody; PSMA, with PE-conjugated PSMA antibody; E/T = 1:1, *n* = 3 biologically independent samples. Data are presented as mean values +/- SD; two-way ANOVA with Sidak's multiple-comparisons test. Source data are provided as a Source Data file.

Supplementary Fig. 4

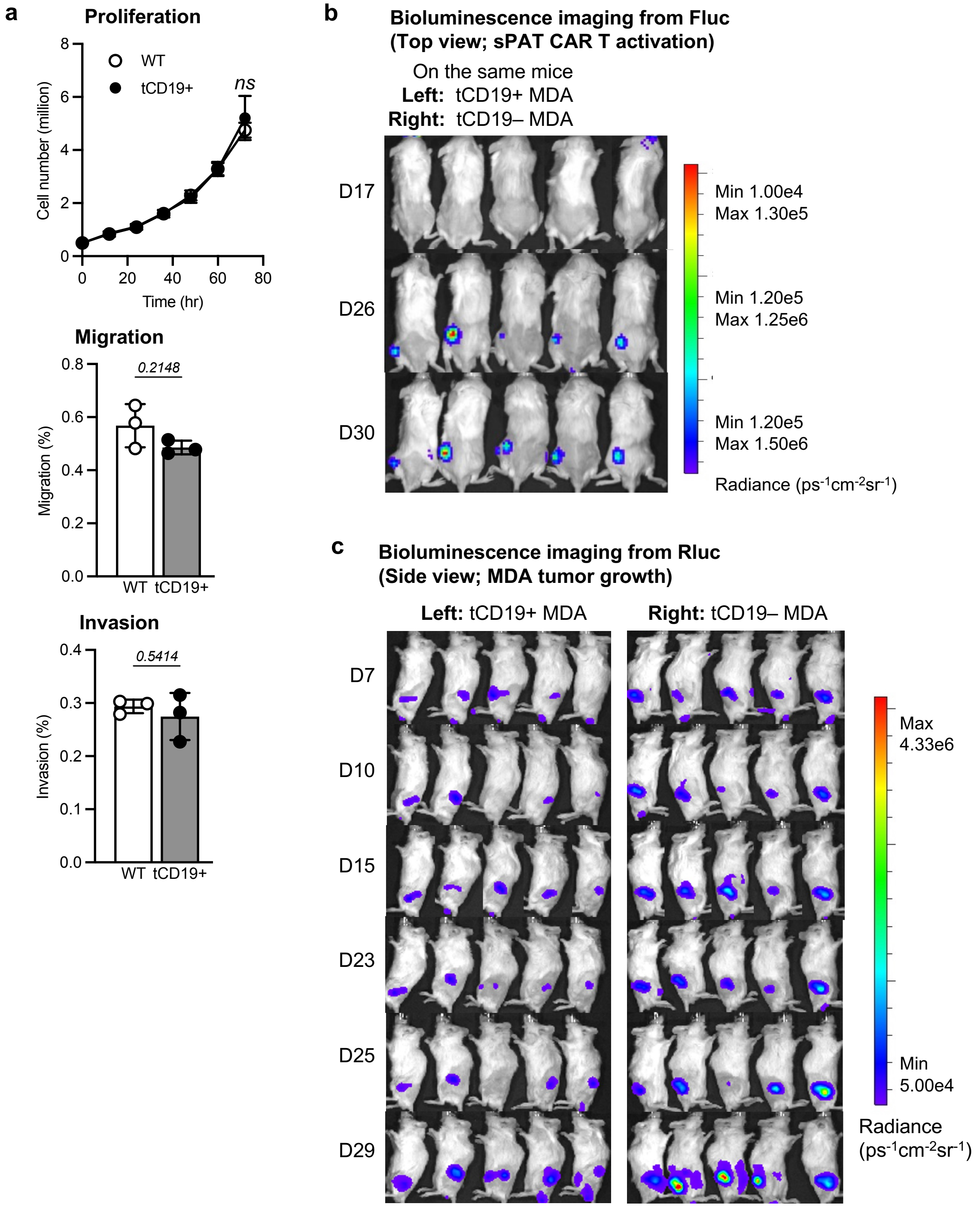

Supplementary Figure 4. sPAT CAR T cells showed homing, activation, and cytotoxicity against target tumor region. (a) tCD19 expression does not alter proliferation, migration, or invasion of MDA-MB-231 cells. Growth curves show no significant difference (data are presented as mean values ± SD, n = 3 biologically independent samples; two-way ANOVA with Sidak's test). Transwell migration and Matrigel invasion at 16 h are plotted as % of input (data are presented as mean values ± SD, *n* = 3 biologically independent samples; unpaired Student's two-tailed t-test). **(b)** NOD/SCID/IL2rγ^{null} (NSG) mice (eight weeks old, female, 5 per group) were subcutaneously injected with 'tCD19+ MDA' cells on one flank and 'tCD19− MDA' cells on the other flank. Engineered T cells with anti-CD19 synNotch and inducible PEbody PAT CAR-P2A-Fluc cassette (enlarged panel) were introduced via tail vein. Bioluminescence images of Fluc showing T cell homing and activation (top view showing both the left and right sides of the mice). **(c)** Bioluminescence images of Rluc showing tumor growth in mice. The left (tCD19+ MDA tumor) and right (tCD19− MDA tumor) sides of the mice were shown respectively. Source data are provided as a Source Data file.

Supplementary Fig. 5

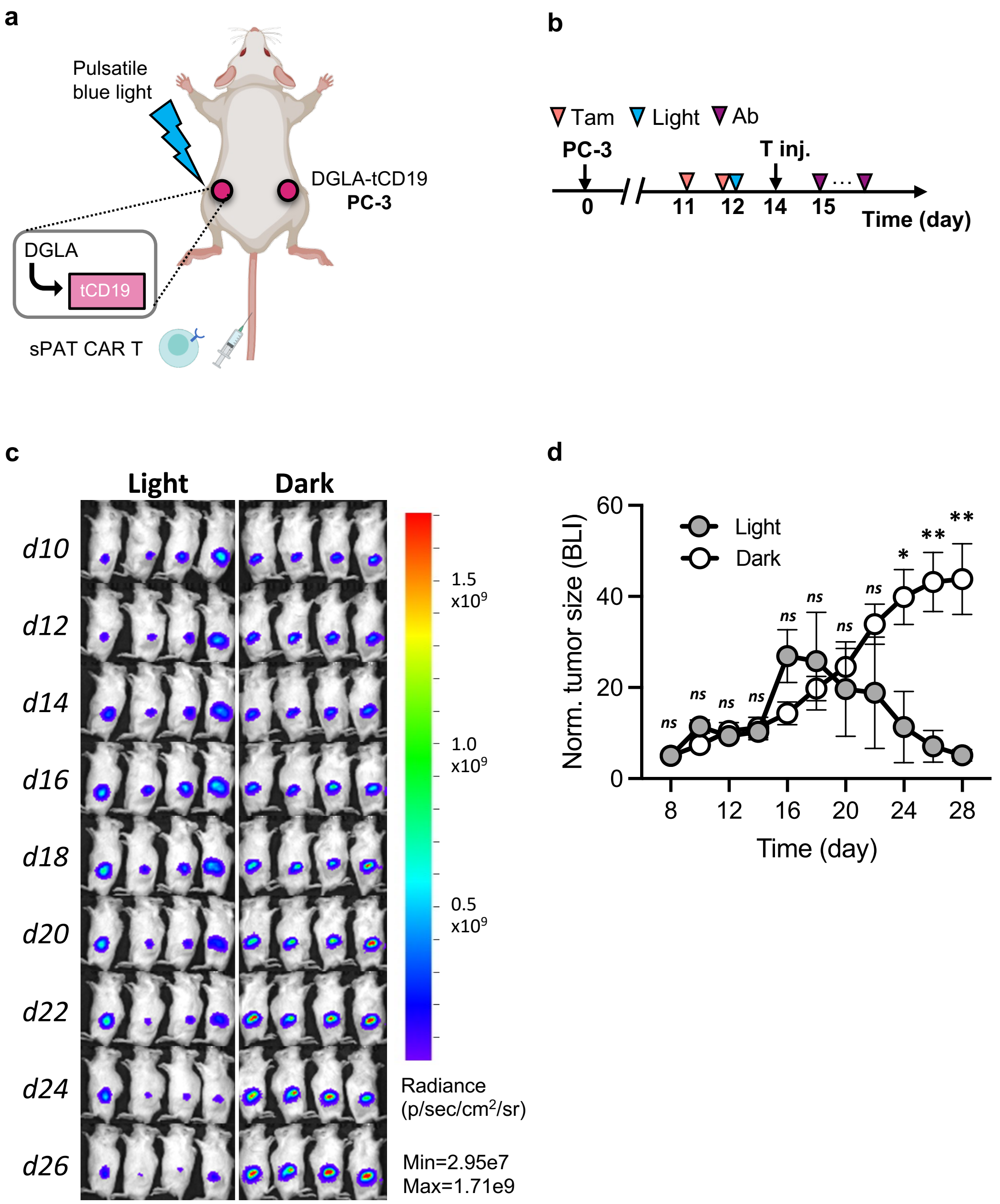

Supplementary Figure 5. DGLA-sPAT CAR T immunotherapy in vivo using a PC-3 model. (a) Schematic of the bilateral PC-3 (PSMA+) tumor model with DGLA-regulated tCD19. The target-side tumor received pulsatile blue-light stimulation; the contralateral tumor remained dark. sPAT CAR T cells were infused via tail vein. NSG mice, 8-week-old, male, 4 per group. Created in BioRender. Guo, T. (2026) https://BioRender.com/0p8wfjp. **(b)** Treatment timeline indicating tumor inoculation (PC-3),

tamoxifen (Tam), light stimulation (Light), T-cell injection (T inj.), and antibody administration (anti-PSMA-PE, Ab). **(c)** Bioluminescence imaging (BLI) of tumor burden under Light versus Dark conditions. (d) Comparison of tumor growth (BLI) between Light and Dark groups (data are presented as mean values +/- SEM, $n=4$ biologically independent animals). Two-way ANOVA with Sidak's multiple-comparisons test; *: $p < 0.05$, **: $p < 0.01$; ns, not significant. Source data are provided as a Source Data file.

Supplementary Fig. 6

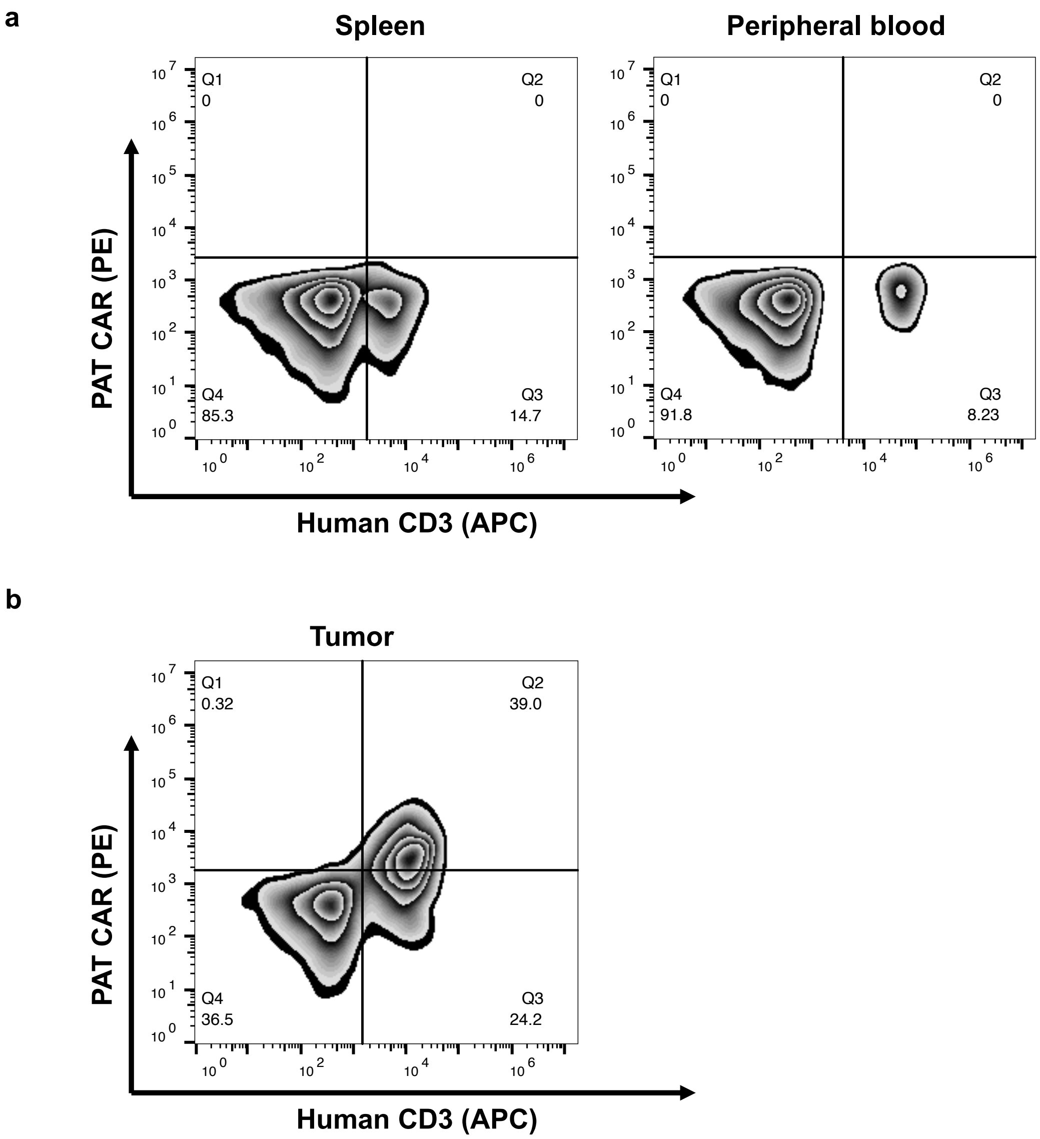

Supplementary Figure 6. **Ex vivo flow cytometry detection of sPAT CAR T cells in mouse tissues.** **(a)** Representative contour plots of single-cell suspensions from spleen and peripheral blood stained for human CD3 (APC) vs PEbody PAT CAR (PE). **(b)** Representative plot from tumor. Quadrants indicate $CD3^+/CAR^+$ (Q2), $CD3^+/CAR^-$ (Q3), $CAR^+/CD3^-$ (Q1), and double negative (Q4) populations; numbers denote percentages of total events shown. Source data are provided as a Source Data file.

Supplementary Fig. 7

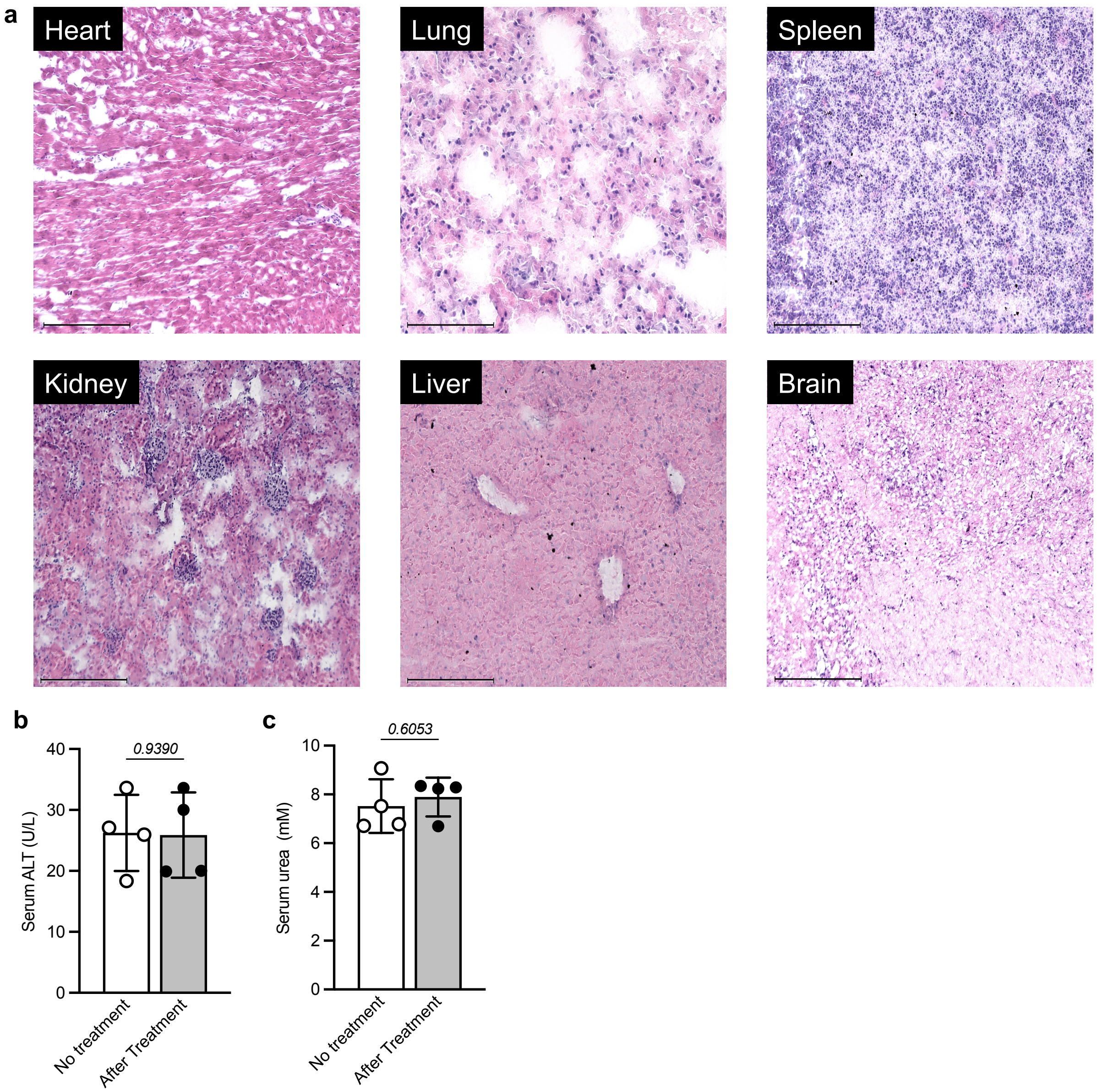

Supplementary Figure 7. Histopathology study and functional tests after PEbody CAR T treatment. (a) Representative H&E staining of heart, lung, spleen, kidney, liver, and brain collected after PEbody CAR T treatment. Scale bars, 275 μm. **(b)** Serum alanine transaminase (ALT) levels in mice with no treatment versus after treatment (data are presented as mean values +/- SD; n = 4 biologically independent samples). **(c)** Serum urea (BUN) levels in the same cohorts (data are presented as mean

values +/- SD; n = 4 biologically independent samples). Unpaired Student's two-tailed t-test; p values shown above bars; ns, not significant. Source data are provided as a Source Data file.

Supplementary Fig. 8

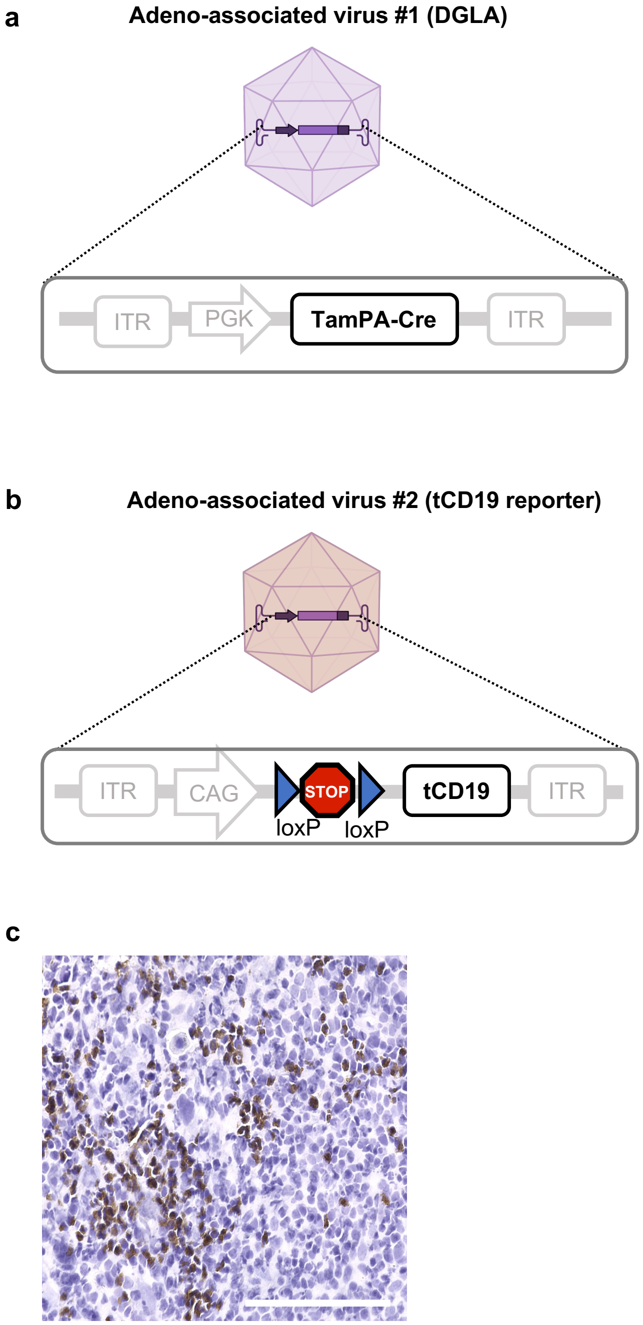

Supplementary Figure 8. Adeno-associated virus (AAV) in situ tumor transduction. (a) AAV vector encoding the DGLA system (TamPA-Cre) driven by constitutive promoter. Created in BioRender. Guo, T. (2026) https://BioRender.com/jthpoac. **(b)** AAV vector encoding the inducible tCD19 reporter (Cre-loxP based). Created in BioRender. Guo, T. (2026) https://BioRender.com/77r4wqz. **(c)** Representative

IHC of tCD19 expression in tumor sections after AAV delivery and light stimulation (DAB, brown; hematoxylin, blue). Scale bar, 125 μm.

Supplementary Fig. 9

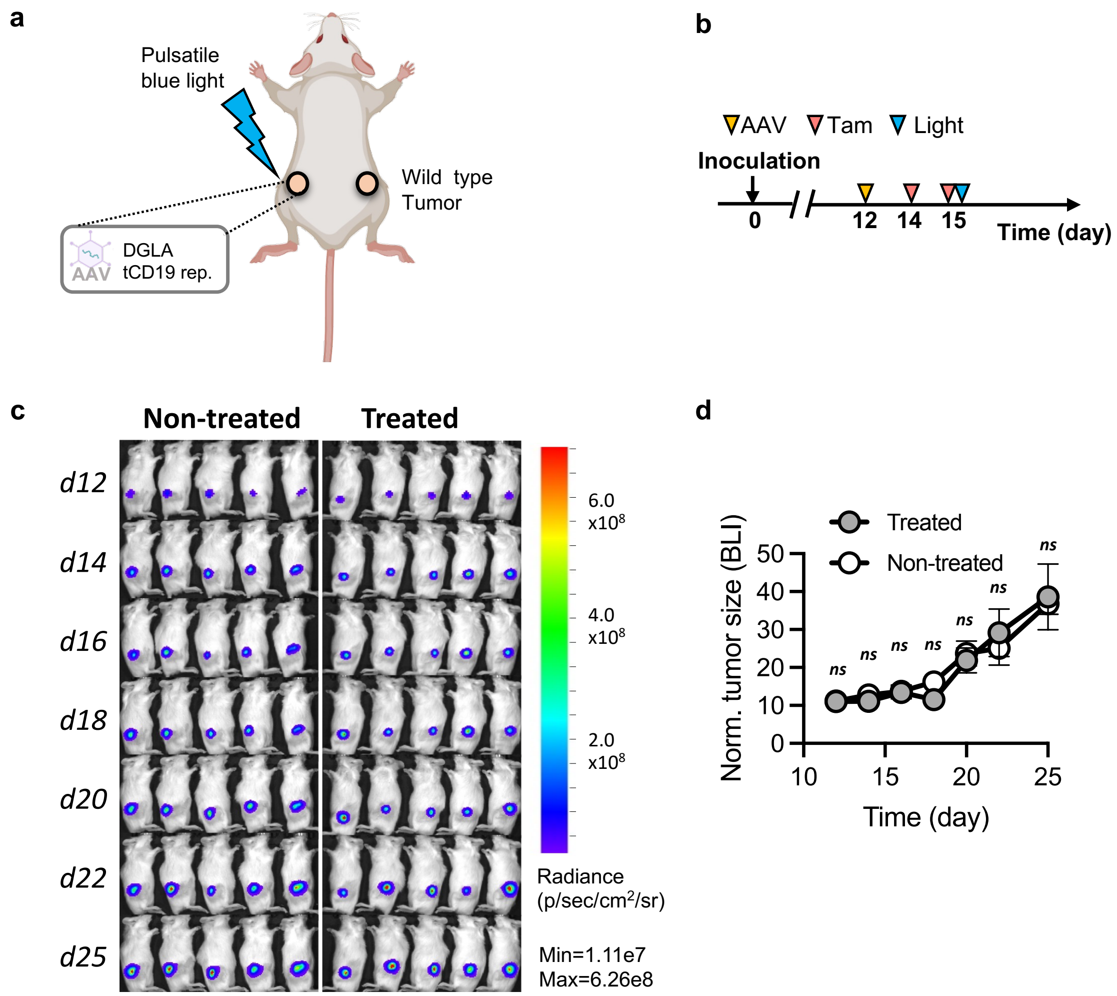

Supplementary Figure 9. Effects of AAV infection and light stimulation on tumor growth. (a) Schematic: one tumor was injected with AAV encoding a DGLA-driven tCD19 reporter and exposed to pulsatile blue light; the contralateral tumor received no AAV, no light. NSG mice, 8-week-old, female, 5 per group. Created in BioRender. Guo, T. (2026) https://BioRender.com/3wonp5g. **(b)** Treatment schedule showing tumor inoculation, AAV administration, tamoxifen (Tam), and light stimulation (Light). **(c)** Bioluminescence imaging (BLI) comparing treated (AAV+/Light) and untreated (AAV-/Dark) tumors. **(d)** Normalized tumor growth by BLI for treated versus untreated tumors. Data are presented as mean values +/- SEM, *n=5* biologically independent animals. Student's two-way ANOVA with Sidak's multiple-comparisons test; ns, not significant. Source data are provided as a Source Data file.

Supplementary Fig. 10

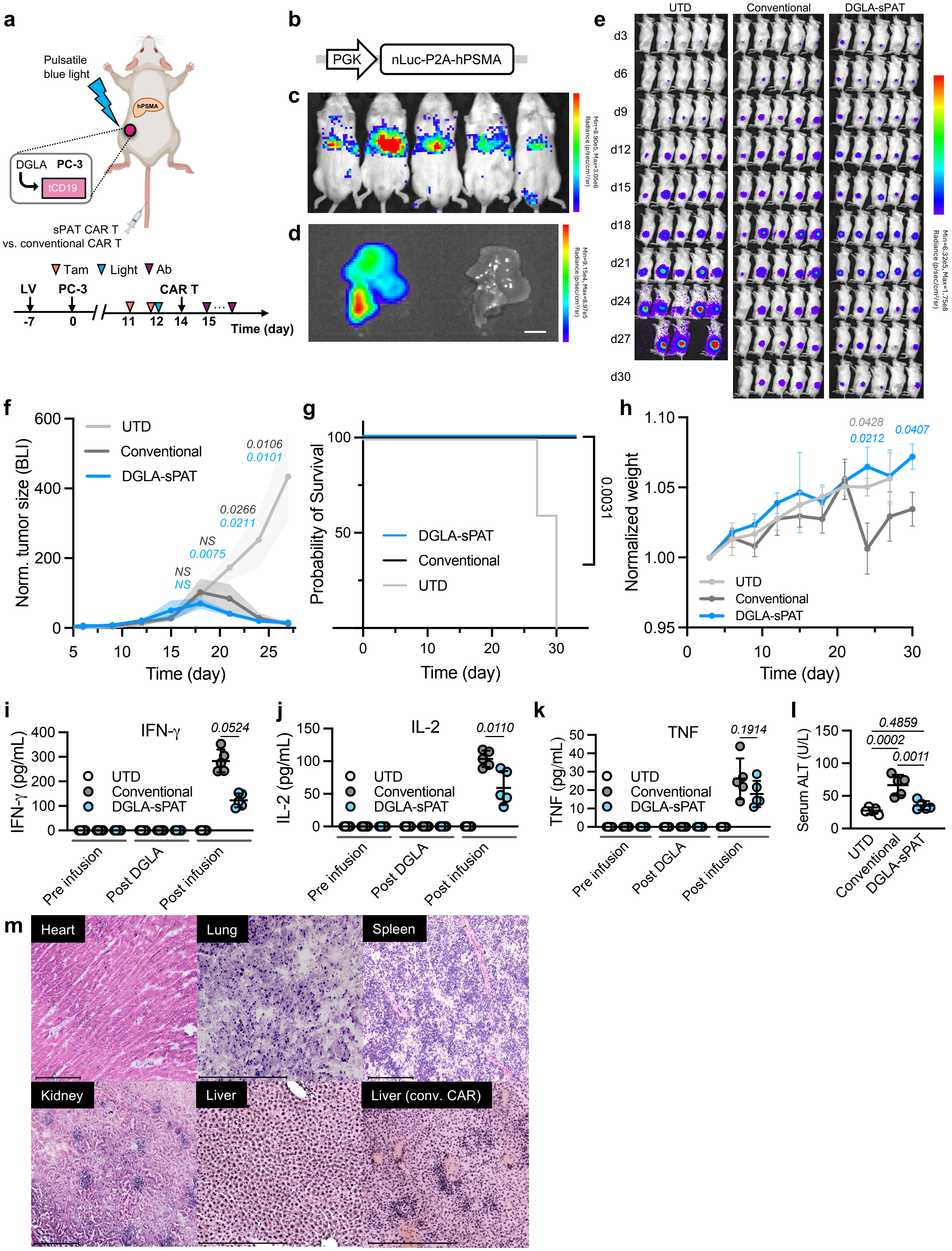

Supplementary Figure 10. DGLA-sPAT CAR T therapy preserves antitumor efficacy while preventing OTOT. (a) Upper: schematic of the in vivo experimental design. Human PSMA (hPSMA) was ectopically expressed in the mouse liver via lentiviral transduction, and mice (8-week-old, NSG, male, 5 per group) bearing a single-flank PSMA$^+$ tumor containing a DGLA-inducible tCD19 cassette were treated with either DGLA-sPAT CAR T cells or conventional PSMA-CAR T cells. Lower: time line for lentiviral transduction (LV), tumor inoculation (PC-3), DGLA-sPAT induction (Tam/Light), anti-PSMA-PE antibody injection (Ab), and infusion of sPAT CAR T or anti-PSMA CAR T cells (CAR T). Created in BioRender. Guo, T. (2026) https://BioRender.com/e99lycs. **(b)** Lentiviral construct encoding nanoLuc luciferase (nLuc) and human PSMA antigen (hPSMA) driven by a constitutive PGK promoter. **(c)** In vivo bioluminescence imaging confirming hepatic expression of human PSMA following lentivirus administration via tail vein. **(d)** Ex vivo bioluminescence imaging of isolated liver tissue further validating liver-localized PSMA expression. Scale bar: 8 mm. **(e)** Bioluminescence imaging (BLI) of tumor (firefly luciferase) comparing untransduced T cells (UTD), conventional anti-PSMA CAR-T cells (Conventional), and DGLA-sPAT CAR T cells (DGLA-sPAT). **(f)** Normalized tumor growth by BLI (data are presented as mean values +/- SEM, $n=5$ biologically independent animals) of different treatment groups. Two-way ANOVA with Sidak's multiple-comparisons test; ns, not significant. P values of Conventional vs. UTD (black) and DGLA-sPAT vs. UTD (blue) are shown at indicated time points. **(g)** Survival analysis. Mantel-cox test; P value of DGLA-sPAT vs. UTD is shown. **(h)** Normalized body weight changes of different treatment groups (data are presented as mean values +/- SEM, $n=5$ biologically independent animals). Student's t test with Holm-Sidak multiple comparison test. P values of DGLA-sPAT vs. Conventional (blue) and UTD vs. Conventional (grey) are shown at indicated time points. **(i-k)** Quantification of systemic cytokine levels (IFN-γ, IL-2, and TNF) of different treatment groups. Data are presented as mean values +/- SD; two-way ANOVA with Sidak's multiple-comparisons test; P values between indicated groups are shown. **(l)** Serum alanine aminotransferase (ALT) levels of different treatment groups. Data are presented as mean values +/- SD; one-way ANOVA with Tukey's multiple-comparisons test; P values between indicated groups are shown. **(m)** Representative H&E

staining of heart, lung, spleen, kidney, and liver from DGLA-sPAT-treated mice, and corresponding liver H&E staining from conventional CAR-T-treated mice. Scale bar: 275 μm. Source data are provided as a Source Data file.

Supplementary Fig. 11

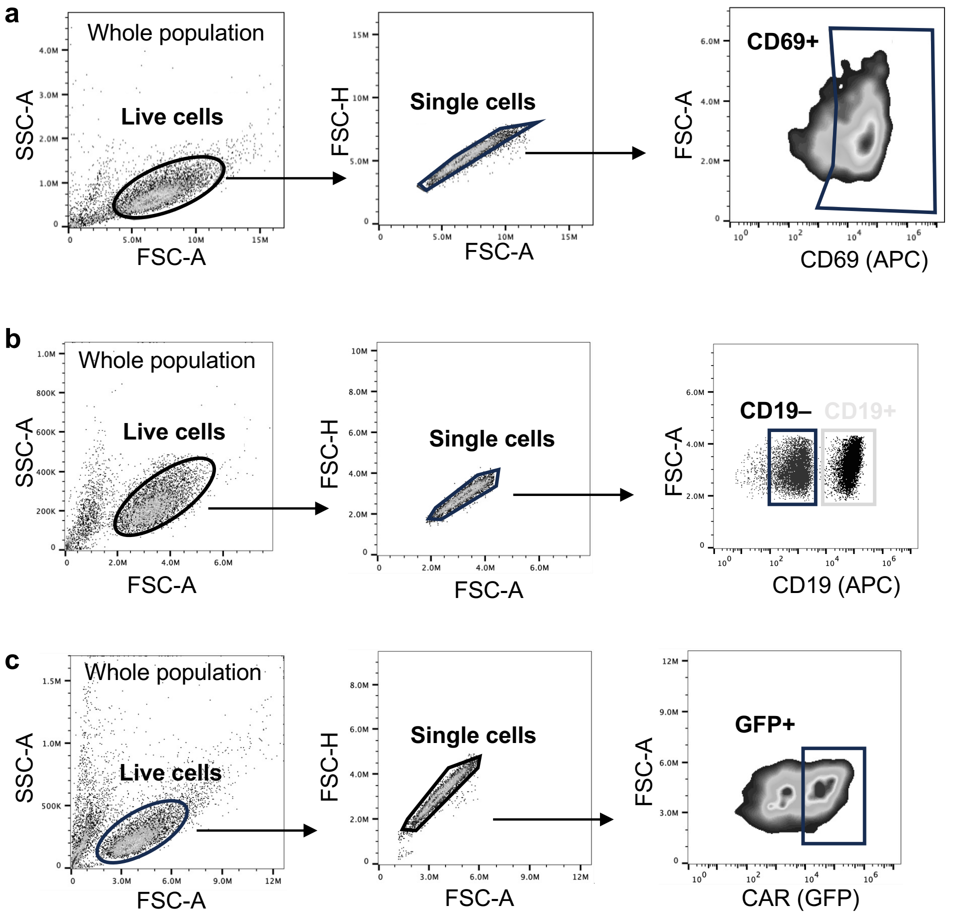

Supplementary Figure 11. Gating strategies used for cell analysis and sorting. (a) Gating strategy for T cell marker analysis, corresponding to Fig. 3f, Supplementary Fig. 1f, Supplementary Fig. 2a-c, Supplementary Fig. 6a-b. **(b)** Gating strategy for CD19-CRISPR knockout cell soring, corresponding to Fig. 2b. **(c)** Gating strategy for T cell transduction analysis and sorting, corresponding to Fig. 3c-e.

Supplementary Table 1. Source information and dilutions of antibodies used in this study.

Antibody	Catalog number	Dilution
PE-conjugated anti-human CD19	BioLegend 302208	1:100
PE-conjugated anti-human CD20	BioLegend 302306	1:100
PE-conjugated anti-human CD38	BioLegend 356604	1:100
PE-conjugated anti-human MUC1	BioLegend 355604	1:100
PE-conjugated anti-human HER2	BioLegend 324406	1:100
PE-conjugated anti-human PD-L1	BioLegend 329706	1:100
PE-conjugated anti-human PSMA	BioLegend 342504	1:100
APC-conjugated anti-human CD69	BioLegend 310910	1:20
APC-conjugated anti-human CD25	BioLegend 302609	1:20
APC-conjugated anti-human CD3	BioLegend 317317	1:20
Mouse IgG1 κ isotype control	BioLegend 400130	1:20
Rat anti-human CD19	Thermo Fisher 53-0194-82	1:500
HRP-conjugated goat anti-rat IgG (H+L)	Thermo Fisher 31470	1:500

Supplementary Table 2. Sequences of oligonucleotides used in this study.

Oligonucleotide	Sequence (5'-3')
gRNA for CD19 CRISPR knockout	CUAGGUCCGAAACAUUCCAC
CD8SP forword primer (includes Kozak sequence)	ctcttcctcatctccgggcctttcggccaccatggctctcccagtg
CD8SP reverse primer	atcagaaacggctgcatgcaggagaagcgctag
PEbody forward primer	tcctgcatgcagccgtttctgatgttccgcgtaag
PEbody reverse primer	aattgcggccgcgctggtacggtagttaatcgag
CD28-41BB-CD3z forward primer	taccgtaccagcgcggccgcaattgaagttatgtatc
CD28-41BB-CD3z reverse primer	cacgcatgttgcaggtgggagttgcttagcgagggggcagggcctg
tCD19 forward primer	atgccacctcctcgcctcctcttc
tCD19 reverse primer	aagatgaagaatgcccacaagggaac
ERT2-CreN-nMag forward primer	atggctggagacatgagagctgccaac
ERT2-CreN-nMag reverse primer	ttctgtttcgcactggaatcccatagag
SV40NLS-pMag-CreC forward primer	cccaagaagaagaggaaagtcggcgga
SV40NLS-pMag-CreC reverse primer	gtccccatcctcgagcagcctcac
