# Supplementary material for: Engineering programmable CAR and antigen pairing via drug-gated light activation
Source: Nat Commun. 2026 Mar 19;17:4252. doi: 10.1038/s41467-026-70855-9 (PMC13168450; doi:10.1038/s41467-026-70855-9)
Supplement: Supplementary file 2 — Reporting Summary [file 41467_2026_70855_MOESM2_ESM.pdf]

nature portfolio

Corresponding author(s): Yingxiao Wang

Last updated by author(s): Feb 18, 2026

Reporting Summary

Nature Portfolio wishes to improve the reproducibility of the work that we publish. This form provides structure for consistency and transparency in reporting. For further information on Nature Portfolio policies, see our Editorial Policies and the Editorial Policy Checklist.

Statistics

For all statistical analyses, confirm that the following items are present in the figure legend, table legend, main text, or Methods section.

n/a	Confirmed	
☐	☒	The exact sample size (*n*) for each experimental group/condition, given as a discrete number and unit of measurement
☐	☒	A statement on whether measurements were taken from distinct samples or whether the same sample was measured repeatedly
☐	☒	The statistical test(s) used AND whether they are one- or two-sided<br>*Only common tests should be described solely by name; describe more complex techniques in the Methods section.*
☒	☐	A description of all covariates tested
☐	☒	A description of any assumptions or corrections, such as tests of normality and adjustment for multiple comparisons
☐	☒	A full description of the statistical parameters including central tendency (e.g. means) or other basic estimates (e.g. regression coefficient) AND variation (e.g. standard deviation) or associated estimates of uncertainty (e.g. confidence intervals)
☐	☒	For null hypothesis testing, the test statistic (e.g. *F*, *t*, *r*) with confidence intervals, effect sizes, degrees of freedom and *P* value noted<br>*Give P values as exact values whenever suitable.*
☒	☐	For Bayesian analysis, information on the choice of priors and Markov chain Monte Carlo settings
☒	☐	For hierarchical and complex designs, identification of the appropriate level for tests and full reporting of outcomes
☒	☐	Estimates of effect sizes (e.g. Cohen's *d*, Pearson's *r*), indicating how they were calculated

Our web collection on statistics for biologists contains articles on many of the points above.

Software and code

Policy information about availability of computer code

Data collection	Living Image 3.0 (Perkin Elmer), Tecan i-control software, BD Accuri C6 Software, SONY SH800 Software.
Data analysis	Image J 2.3.0, Prism 9.5.0 (GraphPad), Living Image 3.0 (Perkin Elmer), FlowJo 10.6.1(BD).

For manuscripts utilizing custom algorithms or software that are central to the research but not yet described in published literature, software must be made available to editors and reviewers. We strongly encourage code deposition in a community repository (e.g. GitHub). See the Nature Portfolio guidelines for submitting code & software for further information.

Data

Policy information about availability of data

All manuscripts must include a data availability statement. This statement should provide the following information, where applicable:

- Accession codes, unique identifiers, or web links for publicly available datasets
- A description of any restrictions on data availability
- For clinical datasets or third party data, please ensure that the statement adheres to our policy

Source data are provided with this paper. The cytotoxicity assays, T-cell activation marker expression, cytokine secretion measurements, bioluminescence imaging, tumor growth and in vivo therapeutic efficacy data, flow cytometry analyses, and histological/serum biochemistry data generated in this study are provided in the Supplementary Information/Source Data file. All data are included in the Supplementary Information or available from the authors, as are unique reagents used in this Article. The raw numbers for charts and graphs are available in the Source Data file whenever possible.

April 2023

Research involving human participants, their data, or biological material

Policy information about studies with human participants or human data. See also policy information about sex, gender (identity/presentation), and sexual orientation and race, ethnicity and racism.

Reporting on sex and gender	No human subjects involved.
Reporting on race, ethnicity, or other socially relevant groupings	No human subjects involved.
Population characteristics	No human subjects involved.
Recruitment	No human subjects involved.
Ethics oversight	No human subjects involved.

Note that full information on the approval of the study protocol must also be provided in the manuscript.

Field-specific reporting

Please select the one below that is the best fit for your research. If you are not sure, read the appropriate sections before making your selection.

[x] Life sciences   [ ] Behavioural & social sciences   [ ] Ecological, evolutionary & environmental sciences

For a reference copy of the document with all sections, see nature.com/documents/nr-reporting-summary-flat.pdf

Life sciences study design

All studies must disclose on these points even when the disclosure is negative.

Sample size	For in vitro experiments, sample sizes of n>=3 for each group were used for statistical analysis purpose. For in vivo experiments, sample sizes of n>=4 for each group were chosen for statistical analysis purpose. The chosen sample sizes were sufficient based on prior experience and publised literature.
Data exclusions	No data were excluded.
Replication	Independent biological repeats were performed to ensure the replicability of results. All attempts at replication were successful.
Randomization	Mice were randomly divided into different treatment groups.
Blinding	The experimenters were not blinded during data collection and analysis. Blinding was not feasible when the same person designed and performed the experiment and analyzed the data. Blinding should not be relevant to the study.

Reporting for specific materials, systems and methods

We require information from authors about some types of materials, experimental systems and methods used in many studies. Here, indicate whether each material, system or method listed is relevant to your study. If you are not sure if a list item applies to your research, read the appropriate section before selecting a response.

Materials & experimental systems

n/a	Involved in the study	
[ ]	[x]	Antibodies
[ ]	[x]	Eukaryotic cell lines
[x]	[ ]	Palaeontology and archaeology
[ ]	[x]	Animals and other organisms
[x]	[ ]	Clinical data
[x]	[ ]	Dual use research of concern
[x]	[ ]	Plants

Methods

n/a	Involved in the study	
[x]	[ ]	ChIP-seq
[ ]	[x]	Flow cytometry
[x]	[ ]	MRI-based neuroimaging

Antibodies

Antibodies used	PE anti-human CD19, Biolegend, Cat# 302208;<br>PE anti-human CD20, Biolegend, Cat# 302306;<br>PE anti-human CD38, Biolegend, Cat# 303506;<br>PE anti-human EGFR, Biolegend, Cat# 352904;

	PE anti-human MUC1, Biolegend, Cat# 355604;<br>PE anti-human HER2, Biolegend, Cat# 324406;<br>PE anti-human PDL1, Biolegend, Cat# 329706;<br>PE anti-human PSMA, Biolegend, Cat# 342504;<br>APC anti-human CD69, Biolegend, Cat# 310910.
Validation	Validated by the manufacturer and other publications.<br>PE anti-human CD19, Biolegend, Cat# 302208: https://www.biolegend.com/en-us/products/pe-anti-human-cd19-antibody-719<br>PE anti-human CD20, Biolegend, Cat# 302306: https://www.biolegend.com/en-us/products/pe-anti-human-cd20-antibody-559<br>PE anti-human CD38, Biolegend, Cat# 303506: https://www.biolegend.com/en-us/products/pe-anti-human-cd38-antibody-746<br>PE anti-human EGFR, Biolegend, Cat# 352904: https://www.biolegend.com/en-us/products/pe-anti-human-egfr-antibody-7432<br>PE anti-human MUC1, Biolegend, Cat# 355604: https://www.biolegend.com/en-us/products/pe-anti-human-cd227-muc-1-antibody-8242<br>PE anti-human HER2, Biolegend, Cat# 324406: https://www.biolegend.com/en-us/products/pe-anti-human-cd340-erbb2-her-2-antibody-3766<br>PE anti-human PD-L1, Biolegend, Cat# 329706: https://www.biolegend.com/en-us/products/pe-anti-human-cd274-b7-h1-pd-l1-antibody-4375<br>PE anti-human PSMA, Biolegend, Cat# 342504: https://www.biolegend.com/en-us/products/pe-anti-human-psma-folh1-antibody-5925<br>APC anti-human CD69, Biolegend, Cat# 310910: https://www.biolegend.com/en-us/products/apc-anti-human-cd69-antibody-1674

Eukaryotic cell lines

Policy information about cell lines and Sex and Gender in Research

Cell line source(s)	Cell lines HEK 293T, MCF-7, Nalm6, PC-3 and MDA-MB-231 were from American Tissue Culture Collection (ATCC, Manassas, VA), with authentication and verification of the absence of mycoplasma contamination.
Authentication	The cell lines were verified by the manufacturer. Routine authentications were performed for all the cell lines listed above via morphology check under microscope and growth rate analyses.
Mycoplasma contamination	All cell lines were tested negative for mycoplasma contamination.
Commonly misidentified lines<br>(See ICLAC register)	No commonly misidentified cell lines were used.

Animals and other research organisms

Policy information about studies involving animals; ARRIVE guidelines recommended for reporting animal research, and Sex and Gender in Research

Laboratory animals	Eight-week-old NOD/SCID/IL2rγnull (NSG) mice were purchased from Jackson Laboratory.
Wild animals	This study did not involve wild animals.
Reporting on sex	Sex was not considered in experiment design and analysis as outcomes were not expected to yield meaningful sex-specific differences, and the objectives of the study were not centered on sex-dependent effects.
Field-collected samples	This study did not involve samples collected from the field.
Ethics oversight	Animal studies were conducted in compliance with all relevant ethical regulations and were approved by the Institutional Animal Care and Use Committee (IACUC) of University of Southern California (Protocol No. #21479). All researchers complied with animal-use guidelines and ethical regulations during animal studies.

Note that full information on the approval of the study protocol must also be provided in the manuscript.

Plants

Seed stocks	NA
Novel plant genotypes	NA
Authentication	NA

Flow Cytometry

Plots

Confirm that:

- [x] The axis labels state the marker and fluorochrome used (e.g. CD4-FITC).
- [x] The axis scales are clearly visible. Include numbers along axes only for bottom left plot of group (a 'group' is an analysis of identical markers).
- [x] All plots are contour plots with outliers or pseudocolor plots.
- [x] A numerical value for number of cells or percentage (with statistics) is provided.

Methodology

Sample preparation	Cells were washed, thoroughly resuspended or filtered to obtain single cell resuspension in PBS before running through flow cytometer. For staining, cells were washed and resuspended in 100 µL wash buffer (PBS + 0.5% BSA) containing the suggested amounts of antibodies, incubated in dark at room temperature for 30 min (or under manufacturers' suggested conditions), and washed three times before being analyzed by flow cytometry.
Instrument	BD Accuri C6, SONY SH800
Software	BD Accuri C6 Software. SONY SH800 Software. FlowJo 10.6.1(BD)
Cell population abundance	The abundance in the post-sort fractions was generally above 95%. The cells were analyzed via flow cytometry every 3-7 days post FACS to ensure purity, and were sorted again if abundance of positive cells dropped to below 90%.
Gating strategy	Gating strategies were demonstrated in Supplementary Figure 11. Gating was based on non-engineered wild type cells. Doublets, dead cells, and non-infected cells were excluded by gating on wild type cells. For flow cytometry on stained cells, gating was based on non- engineered cells with the same staining if not specified in the manuscript.

- [x] Tick this box to confirm that a figure exemplifying the gating strategy is provided in the Supplementary Information.
